# Cell-autonomous megakaryopoiesis associated with polyclonal hematopoiesis in triple-negative essential thrombocythemia

**DOI:** 10.1038/s41598-021-97106-9

**Published:** 2021-09-06

**Authors:** Tadaaki Inano, Marito Araki, Soji Morishita, Misa Imai, Yoshihiko Kihara, Maho Okuda, Yinjie Yang, Masafumi Ito, Satoshi Osaga, Hiroyuki Mano, Yoko Edahiro, Tomonori Ochiai, Kyohei Misawa, Yasutaka Fukuda, Jun Ando, Norio Komatsu

**Affiliations:** 1 Department of Hematology, Juntendo University Graduate School of Medicine, 2-1-1 Hongo, Bunkyo-ku, Tokyo, 113-8421 Japan; 2 Department of Transfusion Medicine and Stem Cell Regulation, Juntendo University Graduate School of Medicine, 2-1-1 Hongo, Bunkyo-ku, Tokyo, 113-8421 Japan; 3 Laboratory for the Development of Therapies against MPN, Juntendo University Graduate School of Medicine, 2-1-1 Hongo, Bunkyo-ku, Tokyo, 113-8421 Japan; 4 Department of Advanced Hematology, Juntendo University Graduate School of Medicine, 2-1-1 Hongo, Bunkyo-ku, Tokyo, 113-8421 Japan; 5 Leading Center for the Development and Research of Cancer Medicine, Juntendo University Graduate School of Medicine, 2-1-1 Hongo, Bunkyo-ku, Tokyo, 113-8421 Japan; 6 Institute for Environmental and Gender-Specific Medicine, Juntendo University Graduate School of Medicine, 2-1-1, Tomioka, Urayasu-city, Chiba 279-0021 Japan; 7 Department of Pathology, Japanese Red Cross Nagoya First Hospital, Aichi, Japan; 8 Clinical Research Management Center, Nagoya City University Hospital, Aichi, Japan; 9 National Cancer Center Research Institute, Tokyo, Japan; 10 Department of Cellular Signaling, Graduate School of Medicine, The University of Tokyo, Tokyo, Japan

**Keywords:** Platelets, Myeloproliferative disease, Myeloproliferative disease, Haematopoietic stem cells

## Abstract

A subset of essential thrombocythemia (ET) cases are negative for disease-defining mutations on *JAK2*, *MPL*, and *CALR* and defined as triple negative (TN). The lack of recurrent mutations in TN-ET patients makes its pathogenesis ambiguous. Here, we screened 483 patients with suspected ET in a single institution, centrally reviewed bone marrow specimens, and identified 23 TN-ET patients. Analysis of clinical records revealed that TN-ET patients were mostly young female, without a history of thrombosis or progression to secondary myelofibrosis and leukemia. Sequencing analysis and human androgen receptor assays revealed that the majority of TN-ET patients exhibited polyclonal hematopoiesis, suggesting a possibility of reactive thrombocytosis in TN-ET. However, the serum levels of thrombopoietin (TPO) and interleukin-6 in TN-ET patients were not significantly different from those in ET patients with canonical mutations and healthy individuals. Rather, CD34-positive cells from TN-ET patients showed a capacity to form megakaryocytic colonies, even in the absence of TPO. No signs of thrombocytosis were observed before TN-ET development, denying the possibility of hereditary thrombocytosis in TN-ET. Overall, these findings indicate that TN-ET is a distinctive disease entity associated with polyclonal hematopoiesis and is paradoxically caused by hematopoietic stem cells harboring a capacity for cell-autonomous megakaryopoiesis.

## Introduction

In approximately 80% of patients with essential thrombocythemia (ET), disease-defining mutations such as *JAK2* V617F, *MPL* exon 10, and *CALR* exon 9 are found in a mutually exclusive manner^1^. Any of these mutant gene products induces the constitutive activation of MPL, the thrombopoietin (TPO) receptor, and its downstream molecules, leading to the clonal expansion of hematopoietic stem cells and the cell-autonomous expansion of megakaryocytes, thus causing thrombocytosis^1^. However, approximately 20% of ET cases do not harbor any of these mutations and thus are called triple-negative ET (TN-ET)^2–8^. Studies have shown the dominance of female patients in the TN-ET patient population, while other clinical parameters, such as age, platelet count, and frequency of thrombosis, vary between studies^7,9,10^, perhaps due to the diversity in ethnicities and medical practices in different institutions. Studies have also shown noncanonical mutations in *JAK2* or *MPL* in a subset of patients with TN-ET. However, noncanonical mutants exhibit a subtle capacity to activate MPL signaling^11,12^, leaving the pathogenesis of TN-ET ambiguous. Here, we performed a single institution study to investigate the clinical and biological features of TN-ET.

## Results

### Younger females are dominant in the TN-ET patient population with no incidence of thrombotic events or progression to fibrosis

**Figure 1:**
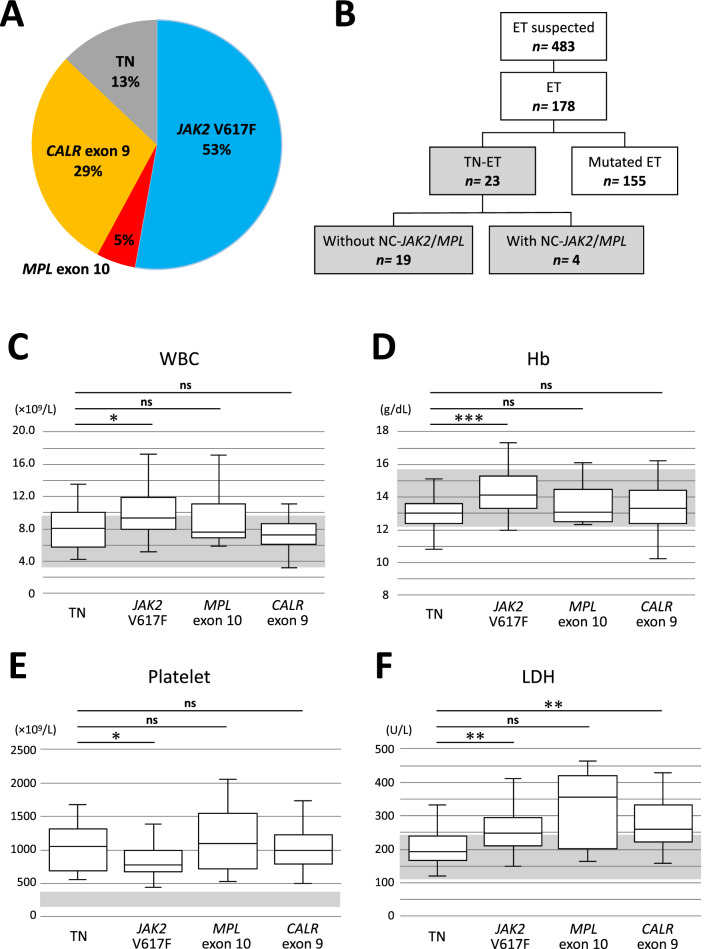
Comparison of clinical parameters between the TN-ET patients and mutated ET patients. (**A**) Frequencies of driver mutations in the ET patients in our cohort. (**B**) A diagram presenting the mutation profiles of the TN-ET patients. NC-*JAK2*/*MPL*: noncanonical *JAK2* and *MPL* mutation. The WBC count (**C**), Hb value (**D**), platelet count (**E**), and LDH level (**F**) for the patients classified based on driver mutation status are shown. Gray highlight shows normal range. * < 0.05, ** < 0.01, *** < 0.001, ns: not significant.

Based on WHO 2016 criteria, 178 patients were defined as having ET, and 13% (n = 23) of them were defined as having TN-ET. Statistical analysis was performed based on the clinical data of the patients grouped by their driver mutation status (Fig. 1A,B; Table 1). The TN-ET patients were mostly females (78.3%) and younger (median age of 36.0 years) than those in the other groups (Table 1). These results suggest that TN-ET is biologically different from ET harboring driver mutations (hereinafter referred to as mutated ET).

**Table 1:** Clinical characteristics of the ET patients grouped by driver mutation status.

	Triple negative	*JAK2* V617F	*MPL* exon 10	*CALR* exon 9	P value							
						TN vs *JAK2*	TN vs *MPL*	TN vs *CALR*	*JAK2* vs *MPL*	*JAK2* vs *CALR*	*MPL* vs *CALR*	
N	23	94	9	52								
Age median [IQR]	36.00 [27.00, 69.00]	59.00 [45.00, 69.00]	51.00 [43.00, 76.00]	47.00 [33.75, 57.25]	**0.006**	0.102	0.721	0.924	0.922	**0.008**	0.919	*
Sex male/female, n (%)	5/18 (21.7/78.3)	43/51 (45.7/54.3)	5/4 (55.6/44.4)	17/35 (32.7/67.3)	0.087							^‡^
WBC (×10^9^/L) median [IQR]	8.1 [5.7, 10.0]	9.3 [8.0, 11.8]	7.6 [7.1, 10.9]	7.3 [6.2, 8.6]	<**0.001**	**0.026**	0.804	0.961	0.611	<**0.001**	0.505	*
Neut (%) mean (SD)	66.86 (10.83)	70.86 (8.07)	70.18 (8.61)	67.21 (7.29)	0.083							^†^
RBC (× 10^12^/L) median [IQR]	4.30 [4.16, 4.74]	4.78 [4.47, 5.12]	4.36 [4.28, 4.74]	4.55 [4.17, 4.76]	<**0.001**	**0.002**	0.897	0.716	0.340	**0.008**	0.998	*
Hb (g/dL) mean (SD)	13.01 (0.95)	14.32 (1.25)	13.53 (1.33)	13.44 (1.39)	<**0.001**	<**0.001**	0.714	0.543	0.278	**0.001**	0.997	^†^
Hct (%) mean (SD)	39.45 (2.85)	43.19 (3.50)	40.41 (3.36)	40.77 (3.93)	<**0.001**	<**0.001**	0.910	0.489	0.145	**0.003**	0.994	^†^
Platelet (×10^9^/L) median [IQR]	1058 [721, 1313]	769 [678, 993]	1091 [720, 1522]	988 [809, 1227]	<**0.001**	**0.034**	0.996	0.988	0.263	**0.001**	0.996	*
EPO (mU/mL) median [IQR]	9.20 [6.70, 16.15]	10.60 [3.60, 14.00]	10.20 [6.50, 14.95]	12.15 [6.38, 19.05]	0.521							*
LDH (U/L) median [IQR]	192.00 [170.00, 237.00]	248.00 [212.00, 295.00]	355.50 [244.75, 405.00]	261.00 [222.00, 332.00]	**0.001**	**0.003**	0.133	**0.004**	0.443	0.763	0.635	*
Fe (μg/dL) mean (SD)	87.67 (27.16)	87.77 (31.47)	80.57 (18.79)	85.58 (23.70)	0.849							^†^
Ferritin (ng/mL) median [IQR]	64.50 [43.00, 102.15]	90.00 [52.25, 146.78]	124.00 [53.00, 157.00]	98.50 [31.50, 147.50]	0.800							*
MF grade 0/1, n (%)	18/0 (100.0/0.0)	58/27 (68.2/31.8)	5/3 (62.5/37.5)	29/12 (70.7/29.3)	**0.045**	**0.032**	**0.034**	0.061	1.000	1.000	1.000	^‡^
Abnormal karyotype Fav/Unfav/VH, n (%)	21/1/0 (95.5/4.5/0.0)	81/9/0 (90.0/10.0/0.0)	9/0/0 (100.0/0.0/0.0)	44/5/0 (89.8/10.2/0.0)	0.650							^‡^
Thrombotic events presentation, n (%)	0/23 (0.0)	25/94 (26.6)	0/9 (0.0)	7/52 (13.5)	**0.006**	**0.032**	1.000	0.388	0.453	0.397	1.000	^‡^
IPSET-thrombosis score low/Int/high, n (%)	18/5/0 (78.3/21.7/0.0)	0/31/63 (0.0/33.0/67.0)	9/0/0 (100.0/0.0/0.0)	44/5/3 (84.6/9.6/5.8)	–	–	–	–	–	–	–	

*IQR* interquartile range, *Fav* Favorable, *Unfav* Unfavorable, *VH* Very High, *Int* Intermediate.

*Kruskal–Wallis test/Steel–Dwass test, ^†^One-way ANOVA/Tukey's HSD test, ‡Chi-square test/chi-square test with Bonferroni correction.

*P*-values in bold indicate significance (< 0.05).

The blood count data of the TN-ET patients resembled those of the ET patients harboring *MPL* exon 10 or *CALR* exon 9 mutations (Fig. 1C–E). However, a significant decrease in WBC count and hemoglobin (Hb) value and an increase in platelet count were observed in the TN-ET patients compared to the ET patients harboring the *JAK2* V617F mutation (Fig. 1C–E). The serum LDH level was significantly reduced in the TN-ET patients compared to the ET patients harboring *JAK2* V617F and *CALR* exon 9 mutations (Fig. 1F). All TN-ET patients displayed a normal or favorable risk karyotype except for one patient with an unfavorable risk karyotype (Table 1; Table S1)^13^.

**Figure 2:**
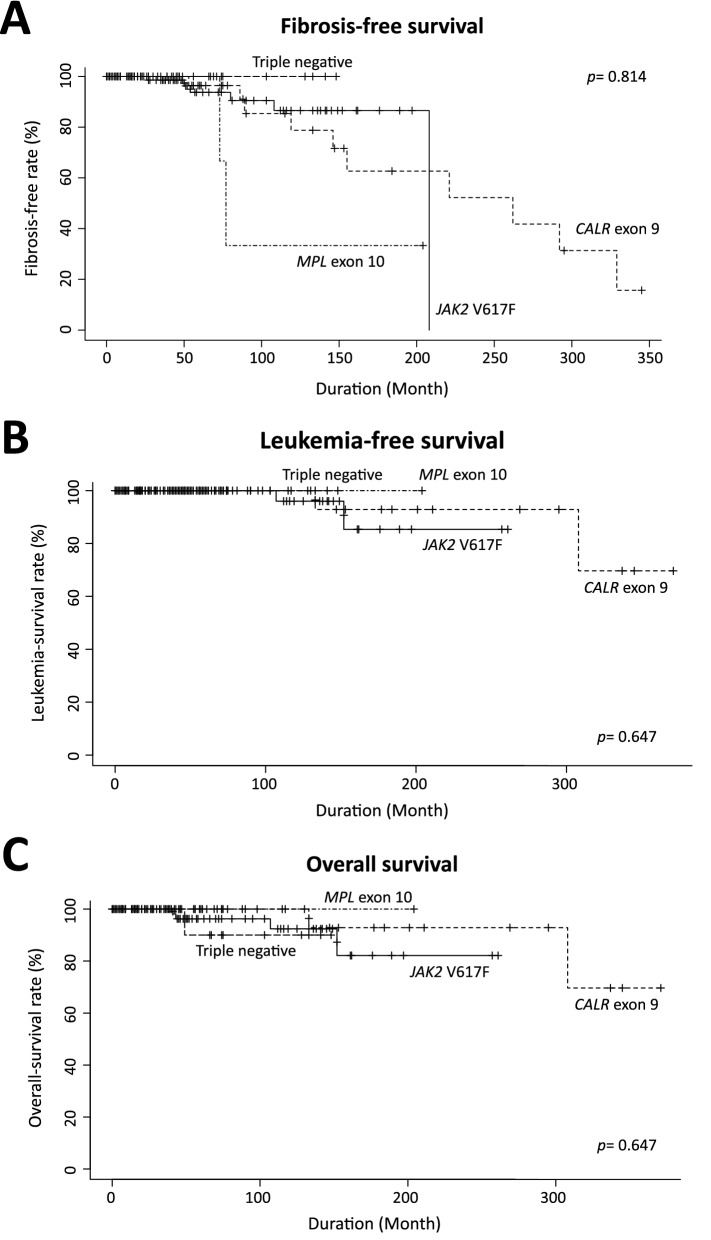
Survival data of the ET patients grouped by driver mutation status. The fibrosis-free survival (**A**), leukemia-free survival (**B**), and overall survival (**C**) of the patients stratified by driver mutation status are shown.

The analysis of clinical events such as thrombosis, progression to fibrosis, and leukemia transformation revealed that the TN-ET patients exhibited none of these events (Table 1; Fig. 2A,B). No significant differences were observed in fibrosis-free survival (FFS), leukemia-free survival (LFS), or overall survival (OS) between the ET groups (Fig. 2A–C), presumably owing to the small size of the cohort. Statistical analysis showed that the number of thrombotic events was significantly reduced in the TN-ET patients compared to the ET patients harboring the *JAK2* V617F mutation (*p* = 0.006, Table 1). Again, these data strongly suggest that TN-ET exhibits clinical features that are different from those of mutated ET.

### Noncanonical JAK2 and MPL mutant exhibits wild-type-equivalent levels of STAT5 activation

**Figure 3:**
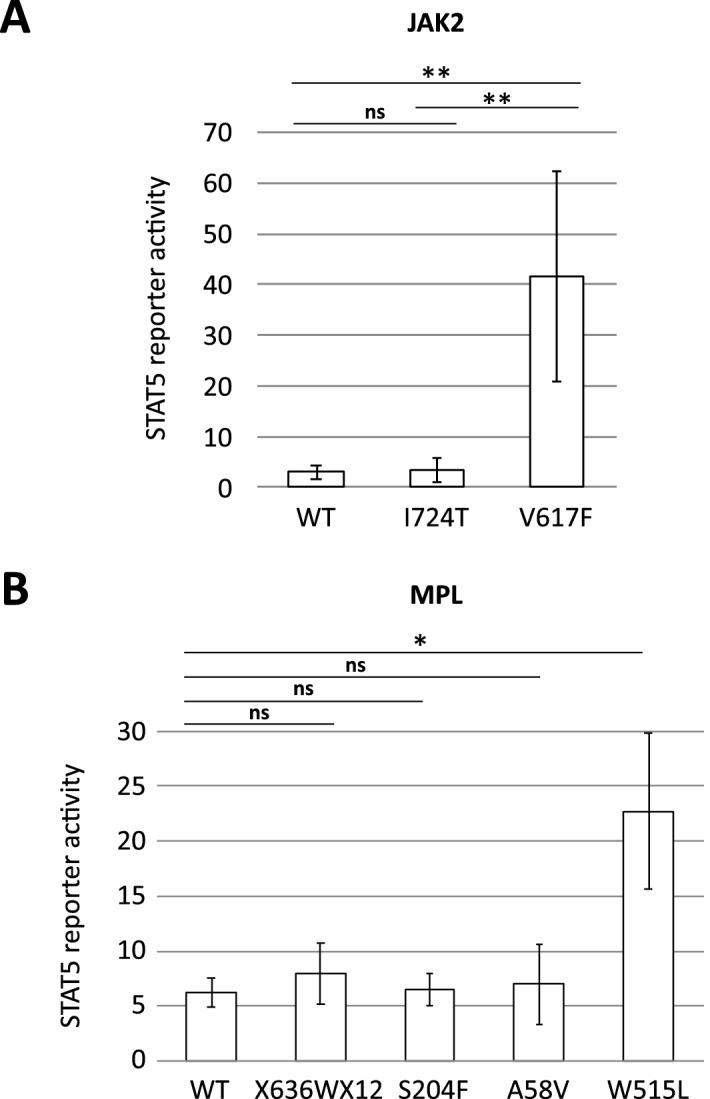
Noncanonical mutants of JAK2 and MPL exhibit wild-type-equivalent levels of STAT5 activation. (**A**,**B**) STAT5 reporter activity determined by luciferase reporter assay. The y-axis indicates values of STAT5 reporter activity adjusted by the internal control. Plasmids encoding JAK2 (**A**) or MPL (**B**) with the indicated mutation were used. Representative data from multiple experiments are presented. * < 0.05, ** < 0.01, ns: not significant.

Because noncanonical mutations in *JAK2* and *MPL* can be found in a subset of triple-negative myeloproliferative neoplasm (TN-MPN) patients^11,12^, we sequenced all exons of *JAK2* and *MPL* for all the TN-ET patients (see Methods) and found 4 noncanonical *JAK2* or *MPL* variants in 4 patients: *JAK2* I724T (germline), *MPL* X636WX12 (germline), *MPL* S204F (somatic), and *MPL* A58V (unknown status due to a lack of germline control) (Fig. 1B; Table S1). To examine the oncogenic properties of these variant gene products, we performed a STAT5 reporter assay (see Methods). We found that, unlike canonical mutants, such as *JAK2* V617F and *MPL* W515L, all noncanonical variants displayed wild-type-equivalent levels of STAT5 activation in the absence of TPO (Fig. 3). In agreement with this result, aside from *MPL* S204F, the noncanonical variants observed are rare variants in the general population (Table S2). In addition, by whole-exome sequencing (WES) analysis of 11 patients with available genomic DNA (gDNA) samples from both peripheral blood and CD3-positive cells, we found that 2 patients harbored somatic mutations (details shown in Table S1). One such patient was found to harbor *MPL* S204F. Nevertheless, no mutation was common in these patients and has been implicated as a driver mutation in MPN.

### Polyclonal hematopoiesis in TN-ET

**Figure 4:**
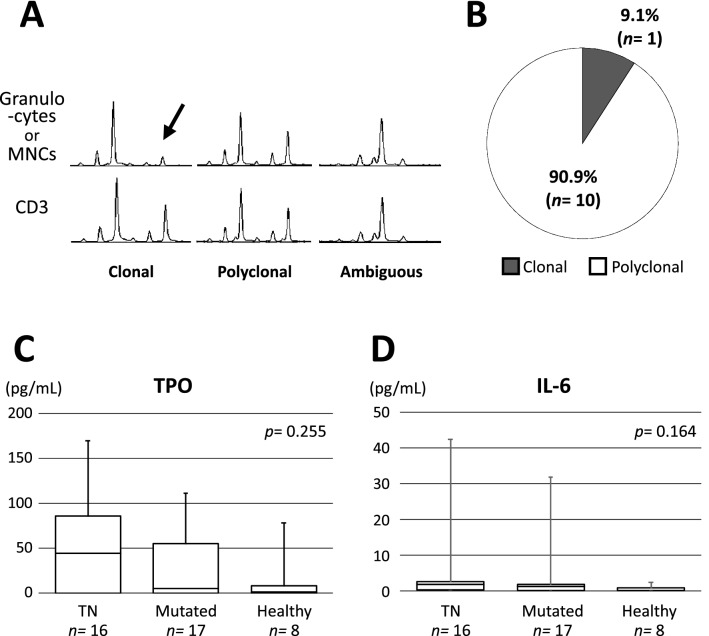
Polyclonal hematopoiesis in the TN-ET patients harboring comparable serum levels of cytokines for megakaryopoiesis. (**A**) Typical profiles of capillary electrophoresis of HpaII-digested gDNA from granulocytes (n = 10) or MNCs (n = 5) and CD3-positive cells. Two HpaII-resistant peaks representing maternal and paternal alleles in polyclonal hematopoiesis. One of these alleles becomes HpaII-sensitive in granulocytes, representing clonal hematopoiesis (arrow). (**B**) A pie chart presenting the frequencies of clonal and polyclonal hematopoiesis judged by the HUMARA assay in TN-ET. Four of 15 patients who exhibited ambiguous patterns in the HUMARA assay (**A**) were excluded from the analysis. Comparison of TPO (**C**) and IL-6 (**D**) concentrations in the serum among the patients with TN-ET, patients with ET harboring driver mutations, and healthy controls.

Because of the considerably low frequency of somatic mutations in the TN-ET patients (Table S1), we suspected that the majority of the TN-ET patients had reactive thrombocytosis with bone marrow (BM) characteristics resembling the MPN. To this end, we performed a *human androgen receptor* (HUMARA) assay to examine the clonality of hematopoietic cells in the TN-ET patients by measuring the degree of skewed methylation on paternal and maternal X-chromosomes^14^. Seventeen of 18 female TN-ET patients had available gDNA from granulocytes (n = 12) or mononuclear cells (MNCs) (n = 5); we assessed their samples, and only one (9.1%) of 11 patients who exhibited judgeable results showed a clonal pattern, while 10 patients (90.9%) showed a polyclonal pattern (Fig. 4B). In contrast, all the patients (n = 3) harboring canonical driver mutations showed a clonal pattern as previously described^15^, and the patients (n = 3) with reactive thrombocytosis showed a polyclonal pattern (data not shown). The frequency (9.1%) of clonal hematopoiesis in TN-ET was within the range expected from a previous study where a higher frequency (14 of 31, 45.2%) of clonal hematopoiesis was observed in elderly females^14^. Nevertheless, despite the histology defining neoplastic features in the BM, most of the TN-ET patients in our cohort exhibited a polyclonal pattern according to the HUMARA assay.

### The levels of serum cytokines were comparable among the TN-ET and ET patients harboring canonical mutations

To search for the cause of thrombocytosis associated with polyclonal hematopoiesis, we examined the levels of serum cytokines, such as TPO^16–19^ and interleukin-6 (IL-6)^16,18,19^, that have been shown to promote platelet production. By analyzing samples from 16 patients with TN-ET, 17 patients with mutated ET, and 8 healthy individuals, we found that the levels of TPO and IL-6 were increased in the patients with TN-ET and mutated ET compared to the healthy controls, but there was no significant difference among the groups, implying that the increases in the levels of TPO and IL-6 do not or only partly promote thrombocytosis in TN-ET (Fig. 4C,D).

### Endogenous megakaryocyte (Mk) colonies were formed from the CD34-positive BM cells of TN-ET patients

**Figure 5:**
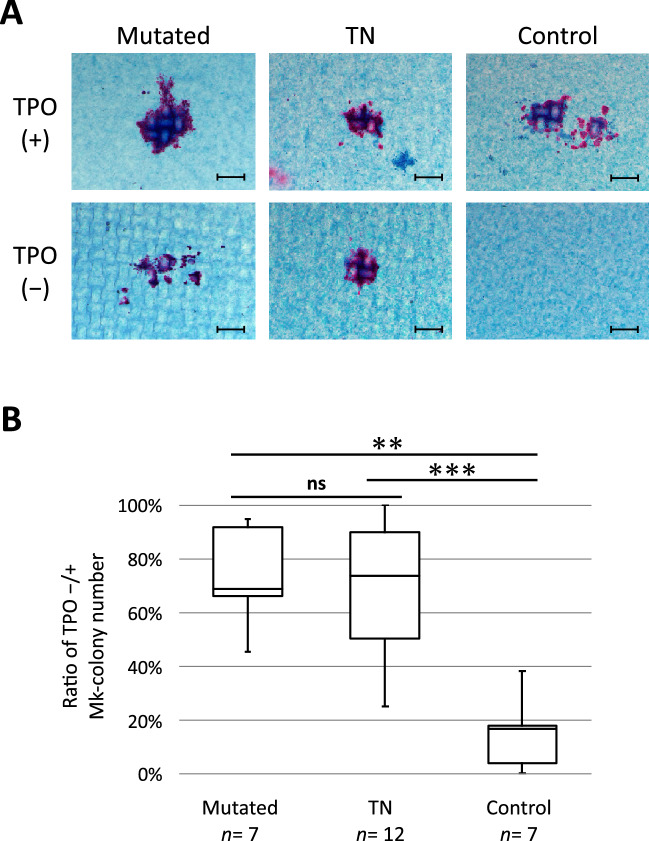
Cell-autonomous megakaryopoiesis in hematopoietic stem/progenitor cells in TN-ET. (**A**) Representative images of megakaryocytic colonies (CD42b-positive) from CD34-positive BM cells from the indicated patients and controls. Scale bar indicates 100 µm. (**B**) Relative ratio of the number of megakaryocytic colonies for the indicated patients and controls formed in the presence and absence of TPO. ** < 0.01, *** < 0.001, ns: not significant.

To examine the capacity of megakaryopoiesis in stem/progenitor cells of the patients with TN-ET, we purified CD34-positive cells from cryopreserved BM cells and cultured them in semisolid media to form Mk colonies (see Methods). Twelve TN-ET, 7 mutated ET, and 7 control samples, including normal (n = 6) and reactive (n = 1) samples, were evaluated. Even in the absence of TPO, Mk colonies formed in TN-ET and mutated ET cell cultures but much less in control cell cultures (Fig. 5A). The capacity to form TPO-independent Mk colonies was determined by the ratio of the number of colonies formed in the absence and presence of TPO and compared between the groups. As shown in Fig. 5B, TN-ET and mutated ET exhibited equivalent capacities to form TPO-independent Mk colonies, and the capacity of these cells was significantly increased compared to that of the control cells. This indicates that despite polyclonal hematopoiesis in TN-ET, CD34-positive cells in TN-ET gain the capacity to promote megakaryopoiesis even in the absence of TPO.

## Discussion

In the present study, we performed an in-depth analysis of the clinical and biological features of TN-ET in a single institution. Statistical analysis of clinical records revealed that young females were predominant in the TN-ET patient population with no incidence of thrombosis and no disease progression to fibrosis and leukemia (Table 1; Fig. 2), strongly suggesting that TN-ET is a disease entity that is biologically distinct from mutated ET. Sequencing analysis failed to identify somatic mutations in the majority of the TN-ET patients, suggesting reactive thrombocytosis associated with polyclonal hematopoiesis in TN-ET. In contrast, no significant elevation of cytokines related to platelet production was observed (Fig. 4). Biological analyses revealed that hematopoietic stem/progenitor cells in TN-ET presented a capacity to promote megakaryopoiesis in the absence of TPO (Fig. 5). Collectively, these findings indicate that TN-ET, which may still be a heterogeneous population, is a distinctive disease entity associated with polyclonal hematopoiesis but is caused in a cell-autonomous manner.

Compared to previous studies with large cohorts, in our cohort, the TN-ET patients (median age of 36.0 years) were younger (ranging between 47 and 59 years)^7,9,10^. Despite such differences that may reflect racial and geographical differences, we observed biological features such as polyclonal hematopoiesis and a low frequency of somatic mutations that resembled those observed among other cohorts. However, it is notable that no thrombotic events were observed in our cohort, while 10–20% of the TN-ET patients in other studies^2,7,9,10^ exhibited thrombosis. While the thrombosis risk determined by the IPSET-thrombosis model^20^ was comparable between TN-ET and *CALR* or *MPL* mutant ET (Table 1), the lack of thrombotic events in TN-ET might indicate the specific biological feature of TN-ET in our cohort.

Consistent with a previous study^11,12,21^, noncanonical *JAK2* and *MPL* mutations were detected by WES or next-generation sequencing (NGS) in a fraction of the TN-ET patients in our cohort. The STAT5 reporter assay revealed that noncanonical mutants of JAK2 and MPL did not show activity similar to that of the canonical mutant proteins but rather exhibited the same level of activity as the wild-type proteins (Fig. 3). Although the noncanonical mutant MPL S204P has been shown to possess a weak gain-of-function property^12^, the present and previous^11^ studies implied that MPL S204F may not be the case. Nevertheless, the low frequency of noncanonical mutations in *JAK2* and *MPL* in TN-ET does not fully explain the pathogenesis of TN-ET.

Polyclonal hematopoiesis was observed in nearly all the TN-ET patients examined, and these patients lack acquired mutations (Table S1). While this suggests the possibility of hereditary thrombocytosis, the platelet count before the diagnosis of all the patients (n = 10) with available blood count data showed no sign of thrombocytosis before diagnosis. Paradoxically, hematopoietic stem/progenitor cells in TN-ET exhibited an oncogenic property in megakaryopoiesis, which may explain the observation of indistinguishable morphology of BM in TN-ET with mutated ET. Based on these facts, we proposed that an environmental cue triggers a persistent epigenetic change in megakaryocytic progenitor cells to confer a capacity to promote megakaryopoiesis in the absence of TPO and results in thrombocytosis. Aberrations in epigenomic regulation not associated with genetic alteration have been shown to promote uncontrolled cell proliferation^22^. Such epigenomic changes may be reversible, and in fact, we observed spontaneous regression of thrombocytosis in one TN-ET patient diagnosed with thrombocytosis 1.5 years previously. Further analyses are required to better understand the pathogenesis of TN-ET, which should lead to the establishment of an appropriate treatment strategy against TN-ET.

In conclusion, we performed in-depth analysis of TN-ET and found that most of the TN-ET patients exhibited polyclonal hematopoiesis with no acquired mutation and no sign of hereditary thrombocytosis. Despite the possibility of reactive thrombocytosis, BM specimens exhibited the features of ET, and hematopoietic stem cells from TN-ET patients showed a capacity for cell-autonomous megakaryopoiesis, the hallmark of ET^23^. Based on these data, we propose that TN-ET, which may still be a heterogeneous population, is a biologically distinctive disease entity; thus, a different treatment strategy may need to be considered from that for mutated ET.

## Methods

### Patients

A total of 483 patients in the Hematology Department, Juntendo University Hospital who were suspected to have ET were analyzed and defined based on the WHO 2016 criteria^24^. Those who were suspected to have familial MPNs were excluded from this study. Preparation of gDNA and analysis of *JAK2* V617F, *MPL* exon 10 and *CALR* exon 9 mutations were performed as described previously^6,25–27^. Histological analysis of BM was performed as described previously^28^. TN-ET was defined by the bone marrow morphology and by evidence of persistent thrombocytosis (> 450 × 10^9^/L for more than six months for all except one who was only followed up for two months) with no potential cause of reactive thrombocytosis. This study was conducted in accordance with the Helsinki Declaration of 1975 and approved by the ethics committee of the School of Medicine, Juntendo University (IRB#2013020). Written informed consent was obtained prior to the use of samples and the collection of clinical records.

### Exon analysis

All exons of *JAK2* and *MPL* were sequenced as described previously^6^. Whole-exome sequencing was performed as described previously^29^. ClinVar^30^, gnomAD (https://gnomad.broadinstitute.org), and ToMMo-4.7KJPN in the Japanese Multi Omics Reference Panel (jMorp) (https://jmorp.megabank.tohoku.ac.jp/202001/) were used to search the frequencies of identified *JAK2* and *MPL* noncanonical mutations in various races (Table S2).

### STAT5 reporter assay

A STAT5 reporter assay was performed as described previously^31^. cDNA of *JAK2* and *MPL* variants were subcloned into pcDNA3.1 plasmids, which were transfected and expressed in HEK293T cells. MPL was coexpressed with wild-type JAK2.

### Statistics

Blood cell counts and biochemical parameters at the first visit prior to the treatment were analyzed. The durations for the development of fibrosis and leukemia were defined from the date of the first visit to that of the diagnosis of grade 2 or 3 fibrosis and to that of the detection of > 20% blasts in the peripheral blood or BM, respectively. Risk stratification according to the chromosome karyotype was defined according to a previous study^13^. The Kruskal–Wallis test/Steel–Dwass test (Table 1; Figs. 1C,E and F, 4C,D, 5B), one-way ANOVA/Tukey’s HSD test (Table 1; Fig. 1D), chi-square test/chi-square test with Bonferroni correction (Table 1), log-rank test (Fig. 2), and Student’s t test (Fig. 3) were used for statistical analysis. *P* values < 0.05 were considered to indicate statistical significance.

### X-chromosome inactivation analysis

Peripheral blood mononuclear cells (PB-MNCs) and granulocytes (PB-Gs) were collected with Lymphosep Lymphocyte Separation Medium (MP Biomedicals) and Lymphocyte Separation Solution (Nacalai Tesque), respectively. CD3-positive cells were collected with the EasySep™ Human CD3 Positive Selection Kit II (STEMCELL Technologies) and expanded in complete medium supplemented with GTS™ OpTmizer™, CTS™ OpTmizer™ (Gibco™), L-glutamine (Gibco™), and human IL-2 (PeproTech). Genomic DNA was purified with the Gentra Puregene Blood Kit (QIAGEN). HUMARA assays, including fragment analysis by capillary electrophoresis using GeneMapper® software, were performed as previously described^14,32^.

### Determination of the serum concentrations of TPO and IL-6

Serum samples were prepared by centrifugation at 1000* g* for 15 min at room temperature and stored in a − 80 °C freezer until examination. To determine the levels of human TPO and IL-6, ELISAs were performed with the Human Thrombopoietin Quantikine ELISA Kit (R&D Systems) and the IL-6 Human Instant ELISA Kit (Invitrogen), respectively, according to the manufacturer’s instructions.

### Human megakaryocyte colony formation assay

A Mk colony formation assay was performed as described previously^33,34^ with minor modifications. CD34-positive cells were purified from cryopreserved bone marrow mononuclear cells (BM-MNCs) using CD34 MicroBead Kit UltraPure (MACS Miltenyi Biotec). CD34-positive cells (5000) were cultured in a chamber (two-chamber slide, Matsunami) with serum-free collagen medium in the presence of 20 ng/mL human IL-3 (hIL-3) (Miltenyi Biotec) and human IL-6 (hIL-6) (Peprotech) and in the presence or absence of 50 ng/mL human thrombopoietin (hTPO) (Kyowa Kirin). The cultures were incubated at 37 °C in a humidified atmosphere of 5% CO_2_ in air for 12–14 days. Fixed cells were stained with anti-CD41 antibody (Clone HIP8, STEMCELL), biotin-conjugated anti-mouse IgG (Dako), streptavidin-conjugated alkaline phosphatase (Vector Laboratory), SIGMA FAST Red TR/naphthol AS-MX alkaline phosphatase substrate (Sigma), and Evans Blue (FUJIFILM Wako). BM-MNCs collected from lymphoma patients without BM infiltration and reactive thrombocytosis patients were used as negative controls.

## Supplementary Information

Supplementary Tables.

## Data Availability

Exome sequencing data is deposited in the National Bioscience Database Center (NBDC; https://biosciencedbc.jp/en/).
