# Author Correction: Cell-autonomous megakaryopoiesis associated with polyclonal hematopoiesis in triple-negative essential thrombocythemia

**DOI:** 10.1038/s41598-022-04977-7

**Published:** 2022-01-17

**Authors:** Tadaaki Inano, Marito Araki, Soji Morishita, Misa Imai, Yoshihiko Kihara, Maho Okuda, Yinjie Yang, Masafumi Ito, Satoshi Osaga, Hiroyuki Mano, Yoko Edahiro, Tomonori Ochiai, Kyohei Misawa, Yasutaka Fukuda, Jun Ando, Norio Komatsu

**Affiliations:** 1 Department of Hematology, Juntendo University Graduate School of Medicine, 2-1-1 Hongo, Bunkyo-ku, Tokyo, 113-8421 Japan; 2 Department of Transfusion Medicine and Stem Cell Regulation, Juntendo University Graduate School of Medicine, 2-1-1 Hongo, Bunkyo-ku, Tokyo, 113-8421 Japan; 3 Laboratory for the Development of Therapies against MPN, Juntendo University Graduate School of Medicine, 2-1-1 Hongo, Bunkyo-ku, Tokyo, 113-8421 Japan; 4 Department of Advanced Hematology, Juntendo University Graduate School of Medicine, 2-1-1 Hongo, Bunkyo-ku, Tokyo, 113-8421 Japan; 5 Leading Center for the Development and Research of Cancer Medicine, Juntendo University Graduate School of Medicine, 2-1-1 Hongo, Bunkyo-ku, Tokyo, 113-8421 Japan; 6 Institute for Environmental and Gender-Specific Medicine, Juntendo University Graduate School of Medicine, 2-1-1, Tomioka, Urayasu-city, Chiba 279-0021 Japan; 7 Department of Pathology, Japanese Red Cross Nagoya First Hospital, Aichi, Japan; 8 Clinical Research Management Center, Nagoya City University Hospital, Aichi, Japan; 9 National Cancer Center Research Institute, Tokyo, Japan; 10 Department of Cellular Signaling, Graduate School of Medicine, The University of Tokyo, Tokyo, Japan

Correction to: *Scientific Reports* https://doi.org/10.1038/s41598-021-97106-9, published online 06 September 2021

The original version of this Article contained errors.

As a result of errors in translation of the results from Japanese into English during preparation of the manuscript, some of the values reported in the paper are off - either by one or two orders of magnitude. This is because of incorrect conversion of Japanese symbol which denotes 10^4^ into 10^3^. This affects RBC count in Table 1, and platelet count in Figure 1E, Table 1 and Supplementary Table 1. These errors are now corrected. Additionally, the Authors revised Figure 1C and WBC row in Table 1 to make reporting of the units consistent. In EPO and Fe lines in Table 1 unit was also added. Figure 4 was corrected to remove spurious arrows in panel 4A.

Original Figure 1 is reproduced below for the record.

**Figure 1:**
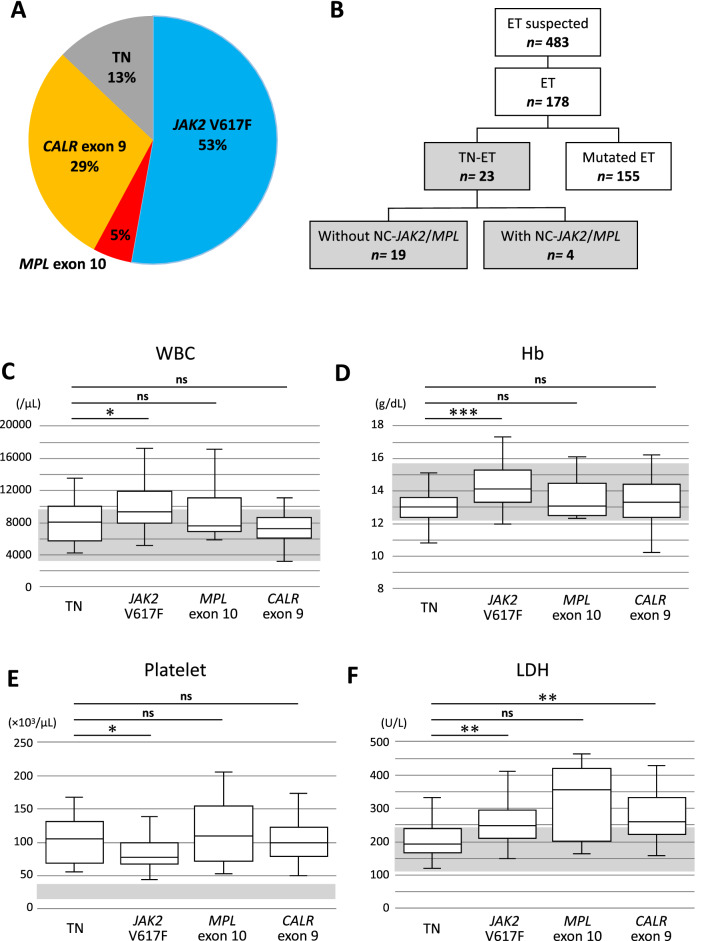
Comparison of clinical parameters between the TN-ET patients and mutated ET patients. (**A**) Frequencies of driver mutations in the ET patients in our cohort. (**B**) A diagram presenting the mutation profiles of the TN-ET patients. NC-*JAK2*/*MPL*: noncanonical *JAK2* and *MPL* mutation. The WBC count (**C**), Hb value (**D**), platelet count (**E**), and LDH level (**F**) for the patients classified based on driver mutation status are shown. Gray highlight shows normal range. * < 0.05, ** < 0.01, *** < 0.001, ns: not significant.

Original Figure 4 is reproduced below for the record.

**Figure 4:**
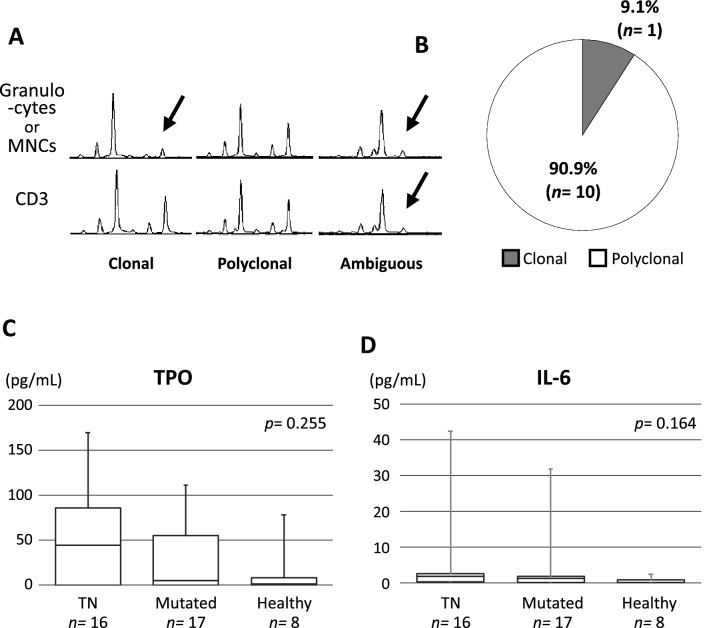
Polyclonal hematopoiesis in the TN-ET patients harboring comparable serum levels of cytokines for megakaryopoiesis. (**A**) Typical profiles of capillary electrophoresis of HpaII-digested gDNA from granulocytes (n = 10) or MNCs (n = 5) and CD3-positive cells. Two HpaII-resistant peaks representing maternal and paternal alleles in polyclonal hematopoiesis. One of these alleles becomes HpaII-sensitive in granulocytes, representing clonal hematopoiesis (arrow). (**B**) A pie chart presenting the frequencies of clonal and polyclonal hematopoiesis judged by the HUMARA assay in TN-ET. Four of 15 patients who exhibited ambiguous patterns in the HUMARA assay (**A**) were excluded from the analysis. Comparison of TPO (**C**) and IL-6 (**D**) concentrations in the serum among the patients with TN-ET, patients with ET harboring driver mutations, and healthy controls.

Original Table 1 is reproduced below for the record.

**Table 1:** Clinical characteristics of the ET patients grouped by driver mutation status.

	Triple negative	*JAK2* V617F	*MPL* exon 10	*CALR* exon 9	P value						
						TN vs *AK2*	TN vs *MPL*	TN vs *CALR*	*JAK2* vs *MPL*	*JAK2* vs *CALR*	*MPL* vs *CALR*
N	23	94	9	52							
Age median [IQR]	36.00 [27.00, 69.00]	59.00 [45.00, 69.00]	51.00 [43.00, 76.00]	47.00 [33.75, 57.25]	**0.006**	0.102	0.721	0.924	0.922	**0.008**	0.919*
Sex male/female, n (%)	5/18 (21.7/78.3)	43/51 (45.7/54.3)	5/4 (55.6/44.4)	17/35 (32.7/67.3)	0.087						‡
WBC (× 10^6^/L) median [IQR]	8100.00 [5700.00, 9950.00]	9300.00 [8000.00, 11,775.00]	7600.00 [7100.00, 10,900.00]	7300.00 [6150.00, 8600.00]	< **0.001**	0.026	0.804	0.961	0.611	< **0.001**	0.505*
Neut (%) mean (SD)	66.86 (10.83)	70.86 (8.07)	70.18 (8.61)	67.21 (7.29)	0.083						^†^
RBC (× 10^9^/L) median [IQR]	430.00 [415.50, 474.00]	477.50 [447.25, 512.00]	436.00 [427.75, 473.50]	454.50 [416.75, 475.75]	< **0.001**	**0.002**	0.897	0.716	0.340	**0.008**	0.998*
Hb (g/dL) mean (SD)	13.01 (0.95)	14.32 (1.25)	13.53 (1.33)	13.44 (1.39)	< **0.001**	< **0.001**	0.714	0.543	0.278	**0.001**	0.997^†^
Hct (%) mean (SD)	39.45 (2.85)	43.19 (3.50)	40.41 (3.36)	40.77 (3.93)	< **0.001**	< **0.001**	0.910	0.489	0.145	**0.003**	0.994^†^
Platelet (× 10^9^/L) median [IQR]	105.80 [72.05, 131.25]	76.90 [67.83, 99.30]	109.10 [72.00, 152.20]	98.80 [80.85, 122.65]	< **0.001**	**0.034**	0.996	0.988	0.263	**0.001**	0.996*
EPO median [IQR]	9.20 [6.70, 16.15]	10.60 [3.60, 14.00]	10.20 [6.50, 14.95]	12.15 [6.38, 19.05]	0.521						*
LDH (U/L) median [IQR]	192.00 [170.00, 237.00]	248.00 [212.00, 295.00]	355.50 [244.75, 405.00]	261.00 [222.00, 332.00]	**0.001**	**0.003**	0.133	**0.004**	0.443	0.763	0.635*
Fe mean (SD)	87.67 (27.16)	87.77 (31.47)	80.57 (18.79)	85.58 (23.70)	0.849						^†^
Ferritin (ng/mL) median [IQR]	64.50 [43.00, 102.15]	90.00 [52.25, 146.78]	124.00 [53.00, 157.00]	98.50 [31.50, 147.50]	0.800						*
MF grade 0/1, n (%)	18/0 (100.0/0.0)	58/27 (68.2/31.8)	5/3 (62.5/37.5)	29/12 (70.7/29.3)	**0.045**	**0.032**	**0.034**	0.061	1.000	1.000	1.000‡
Abnormal karyotype Fav/Unfav/VH, n (%)	21/1/0 (95.5/4.5/0.0)	81/9/0 (90.0/10.0/0.0)	9/0/0 (100.0/0.0/0.0)	44/5/0 (89.8/10.2/0.0)	0.650						‡
Thrombotic events presentation, n (%)	0/23 (0.0)	25/94 (26.6)	0/9 (0.0)	7/52 (13.5)	**0.006**	**0.032**	1.000	0.388	0.453	0.397	1.000‡
IPSET-thrombosis score Low/Int/High, n (%)	18/5/0 (78.3/21.7/0.0)	0/31/63 (0.0/33.0/67.0)	9/0/0 (100.0/0.0/0.0)	44/5/3 (84.6/9.6/5.8)	–	–	–	–	–	–	–

*IQR* interquartile range, *Fav* Favorable, *Unfav* Unfavorable, *VH* Very High, *Int* Intermediate.

*Kruskal–Wallis test/Steel–Dwass test, ^†^One-way ANOVA/Tukey's HSD test, ‡Chi-square test/chi-square test with Bonferroni correction.

*P*-values in bold indicate significance (< 0.05).

Original Supplementary Table 1 is reproduced below as Table 2 for the record.

**Table 2:** Clinical, biological, and genetic characteristics of TN-ET patients.

Patient no.	Age	Sex	Duration of follow up (months)	Outcome	Chromosome karyotype	Platelet^a^ (10^6^/μL)	WES	All exon analysis of *JAK2* and *MPL*	HUMARA assay	TPO (pg/mL)	IL-6 (pg/mL)	Mk colony-forming assay
ET01	81	F	49	Died due to aortic dissection	Normal karyotype	58.2	Not detected	ND	Ambiguous	ND	ND	Cell-autonomous
ET02	74	M	47	Alive	45,X,−Y	158.1	*DNMT3A*, *SRSF7*	ND	NA	100.37	1.43	Cell-autonomous
ET03	20	F	22	Alive	Normal karyotype	56.0	ND	Not detected	Ambiguous	0.01	0.00	ND
ET08	38	F	24	Alive	46,XY,inv(9)(p12q13)	167.4	ND	Not detected	Polyclonal	85.35	0.44	ND
ET10	53	F	66	Alive	Normal karyotype	113.3	*JAK2* I724T (germline)	ND	Ambiguous	11.60	2.76	Cell-autonomous
ET15	16	F	103	Alive	ND	164.3	*MPL* X636WX12 (germline)	ND	Polyclonal	0.00	0.89	ND
ET16	37	F	128	Alive	46,XX,+X	123.4	Not detected	ND	Polyclonal	169.59	2.52	Cell-autonomous
ET17	81	F	45	Alive	Normal karyotype	75.3	ND	Not detected	Clonal	0.00	2.32	Cell-autonomous
ET18	77	M	39	Alive	Normal karyotype	88.2	*MPL* S204F, *ZBTB7A*, *ADAMTS9*, *AGAP2*, *HEPACAM*, *MYPN*, *PDE1C*, *PLCD3*, *TCF3*	ND	NA	ND	ND	Cell-autonomous
ET21	82	M	6	Alive	Normal karyotype	98.7	ND	*MPL* A58V	NA	ND	ND	Cell-autonomous
ET26	34	F	74	Alive	46,XX,t(6;12)(p21;p13)	105.8	Not detected	ND	Polyclonal	60.62	2.07	ND
ET27	27	F	75	Alive	Normal karyotype	113.6	Not detected	ND	Polyclonal	ND	ND	Cell-autonomous
ET28	78	M	133	Alive	45,X.-Y	63.7	ND	Not detected	NA	ND	ND	ND
ET31	18	F	46	Alive	Normal karyotype	93.9	Not detected	ND	Polyclonal	69.27	3.47	ND
ET32	51	F	18	Alive	Normal karyotype	63.1	ND	Not detected	Ambiguous	0.09	20.01	ND
ET33	15	F	43	Alive	Normal karyotype	131.7	Not detected	ND	Ambiguous	52.85	1.86	Cell-autonomous
ET36	63	M	148	Alive	Normal karyotype	130.8	ND	Not detected	NA	ND	ND	ND
ET38	44	F	141	Alive	Normal karyotype	105.9	ND	Not detected	Polyclonal	0.00	0.00	Cell-autonomous
ET39	33	F	17	Alive	Normal karyotype	100.2	ND	Not detected	Polyclonal	0.00	1.54	Cell-autonomous
ET40	66	F	67	Alive	Normal karyotype	153.9	Not detected	ND	Polyclonal	90.45	42.46	ND
ET41	37	F	16	Alive	Normal karyotype	55.6	ND	Not detected	Polyclonal	87.48	0.00	ND
ET45	28	F	2	Alive	Normal karyotype	146.6	ND	Not detected	ND	ND	ND	ND
ET46	24	F	15	Alive	Normal karyotype	68.8	ND	Not detected	Ambiguous	35.69	0.00	Cell-autonomous

WES, whole-exome sequencing; Mk, megakaryocyte; ND, not done; NA, not applicable.

*Platelet counts are those from the first consultation.

Finally, in the Results, under the subheading ‘Noncanonical JAK2 and MPL mutant exhibits wild-type-equivalent levels of STAT5 activation’,

“One such patient was found to harbor MPL S204F in the abovementioned screening of noncanonical JAK2 and MPL mutations.”

now reads

“One such patient was found to harbor MPL S204F.”

These errors do not affect the conclusions of the Article. The Article and the accompanying Supplementary Information file have been corrected.

