# Supplementary material for: Cell-autonomous megakaryopoiesis associated with polyclonal hematopoiesis in triple-negative essential thrombocythemia
Source: Sci Rep. 2021 Sep 6;11:17702. doi: 10.1038/s41598-021-97106-9 (PMC8421373; doi:10.1038/s41598-021-97106-9)
Supplement: Supplementary file 1 — Supplementary Tables. [file 41598_2021_97106_MOESM1_ESM.pdf]

Supportive information

Supplemental Table 1: Clinical, biological, and genetic characteristics of TN-ET patients

Patient No.	Age	Sex	Duration of follow up (months)	Outcome	Chromosome karyotype	Platelet* (10^9/L)	WES	All exon analysis of *JAK2* and *MPL*	HUMARA assay	TPO (pg/mL)	IL-6 (pg/mL)	Mk colony-forming assay
ET01	81	F	49	Died due to aortic dissection	Normal karyotype	582	Not detected	ND	Ambiguous	ND	ND	Cell-autonomous
ET02	74	M	47	Alive	45,X,-Y	1581	*DNMT3A*, *SRSF7*	ND	NA	100.37	1.43	Cell-autonomous
ET03	20	F	22	Alive	Normal karyotype	560	ND	Not detected	Ambiguous	0.01	0.00	ND
ET08	38	F	24	Alive	46,XY,inv(9)(p12q13)	1674	ND	Not detected	Polyclonal	85.35	0.44	ND
ET10	53	F	66	Alive	Normal karyotype	1133	*JAK2* I724T (germline)	ND	Ambiguous	11.60	2.76	Cell-autonomous
ET15	16	F	103	Alive	ND	1643	*MPL* X636WX12 (germline)	ND	Polyclonal	0.00	0.89	ND
ET16	37	F	128	Alive	46,XX,+X	1234	Not detected	ND	Polyclonal	169.59	2.52	Cell-autonomous
ET17	81	F	45	Alive	Normal karyotype	753	ND	Not detected	Clonal	0.00	2.32	Cell-autonomous
ET18	77	M	39	Alive	Normal karyotype	882	*MPL* S204F, *ZBTB7A*, *ADAMTS9*, *AGAP2*, *HEPACAM*, *MYPN*, *PDE1C*, *PLCD3*, *TCF3*	ND	NA	ND	ND	Cell-autonomous
ET21	82	M	6	Alive	Normal karyotype	987	ND	*MPL* A58V	NA	ND	ND	Cell-autonomous
ET26	34	F	74	Alive	46,XX,t(6;12)(p21;p13)	1058	Not detected	ND	Polyclonal	60.62	2.07	ND
ET27	27	F	75	Alive	Normal karyotype	1136	Not detected	ND	Polyclonal	ND	ND	Cell-autonomous
ET28	78	M	133	Alive	45,X.-Y	637	ND	Not detected	NA	ND	ND	ND
ET31	18	F	46	Alive	Normal karyotype	939	Not detected	ND	Polyclonal	69.27	3.47	ND
ET32	51	F	18	Alive	Normal karyotype	631	ND	Not detected	Ambiguous	0.09	20.01	ND
ET33	15	F	43	Alive	Normal karyotype	1317	Not detected	ND	Ambiguous	52.85	1.86	Cell-autonomous
ET36	63	M	148	Alive	Normal karyotype	1308	ND	Not detected	NA	ND	ND	ND
ET38	44	F	141	Alive	Normal karyotype	1059	ND	Not detected	Polyclonal	0.00	0.00	Cell-autonomous
ET39	33	F	17	Alive	Normal karyotype	1002	ND	Not detected	Polyclonal	0.00	1.54	Cell-autonomous
ET40	66	F	67	Alive	Normal karyotype	1539	Not detected	ND	Polyclonal	90.45	42.46	ND
ET41	37	F	16	Alive	Normal karyotype	556	ND	Not detected	Polyclonal	87.48	0.00	ND
ET45	28	F	2	Alive	Normal karyotype	1466	ND	Not detected	ND	ND	ND	ND
ET46	24	F	15	Alive	Normal karyotype	688	ND	Not detected	Ambiguous	35.69	0.00	Cell-autonomous

*Platelet counts are those from the first consultation.
Abbreviations: WES, whole-exome sequencing; Mk, megakaryocyte; ND, not done; NA, not applicable.

Supplemental Table 2: Frequencies of genetic variants of *JAK2* and *MPL* in the general population

Variant	dbSNP id	ClinVar	gnomAD							ToMMo-4.7KJPN
			All	EAS	SAS	AFR	EUR	LAT	FIN	
JAK2 I724T	rs372254348	US	0.0085%	0.0551%	0.0098%	0.0040%	0.0046%	0.0057%	0.0%	0.0600%
MPL X636WX12	rs756235478	no	0.0071%	0.1003%	0.0%	0.0%	0.0%	0.0%	0.0%	0.7400%
MPL A58V	rs6087	US	0.0127%	0.1504%	0.0%	0.0120%	0.0023%	0.0%	0.0%	0.2700%
MPL S204F	-	-	-	-	-	-	-	-	-	-

Abbreviations: US, uncertain significance; EAS, East Asian; SAS, South Asian; AFR, African; EUR, European; LAT, Latino; FIN, Finnish.
